# Reducing risks by transforming landscapes: Cross-scale effects of land-use changes on ecosystem services

**DOI:** 10.1371/journal.pone.0195895

**Published:** 2018-04-24

**Authors:** Giacomo Fedele, Bruno Locatelli, Houria Djoudi, Matthew J. Colloff

**Affiliations:** 1 Center for International Forestry Research (CIFOR), Bogor, West Java, Indonesia; 2 Research Unit Forêts et Sociétés, Centre de Coopération Internationale en Recherche Agronomique pour le Développement (CIRAD), Montpellier, Occitanie, France; 3 Doctoral School ABIES, AgroParisTech, Paris, Île-de-France, France; 4 Center for International Forestry Research (CIFOR), Lima, Peru; 5 Fenner School of Environment and Society, Australian National University, Canberra, Australian Capital Territory, Australia; University of Vermont, UNITED STATES

## Abstract

Globally, anthropogenic environmental change is exacerbating the already vulnerable conditions of many people and ecosystems. In order to obtain food, water, raw materials and shelter, rural people modify forests and other ecosystems, affecting the supply of ecosystem services that contribute to livelihoods and well-being. Despite widespread awareness of the nature and extent of multiple impacts of land-use changes, there remains limited understanding of how these impacts affect trade-offs among ecosystem services and their beneficiaries across spatial scales. We assessed how rural communities in two forested landscapes in Indonesia have changed land uses over the last 20 years to adapt their livelihoods that were at risk from multiple hazards. We estimated the impact of these adaptation strategies on the supply of ecosystem services by comparing different benefits provided to people from these land uses (products, water, carbon, and biodiversity), using forest inventories, remote sensing, and interviews. Local people converted forests to rubber plantations, reforested less productive croplands, protected forests on hillsides, and planted trees in gardens. Our results show that land-use decisions were propagated at the landscape scale due to reinforcing loops, whereby local actors perceived that such decisions contributed positively to livelihoods by reducing risks and generating co-benefits. When land-use changes become sufficiently widespread, they affect the supply of multiple ecosystem services, with impacts beyond the local scale. Thus, adaptation implemented at the local-scale may not address development and climate adaptation challenges at regional or national scale (e.g. as part of UN Sustainable Development Goals or actions taken under the UNFCCC Paris Agreement). A better understanding of the context and impacts of local ecosystem-based adaptation is fundamental to the scaling up of land management policies and practices designed to reduce risks and improve well-being for people at different scales.

## Introduction

Many societies around the world are facing major environmental challenges that are increasingly complex, uncertain, and interconnected [1]. Global drivers of change such as climate change, human population growth, resource use and environmental degradation, urbanization, and economic globalization are exacerbating the vulnerability of people in already fragile contexts. In order to respond to these challenges, people have developed adaptation strategies to reduce risks to livelihoods and maintain well-being. These adaptation strategies can be anticipatory or reactive and include building infrastructure (e.g. for water storage and flood protection), changing social-economic behaviors (e.g. reducing consumption, selling assets and borrowing money), or using natural resources (e.g. improving crop varieties, harvesting forest products and protecting coastal mangroves).

Nature provides benefits to people from ecosystem services, including the mitigation of impacts of natural hazards and strengthening social capacity to respond to environmental change [2,3]. Provisioning services from forests and agroecosystems provide food, energy, water and construction material that help many rural communities around the world to diversify livelihoods and distribute risks [4]. In addition, regulating services, including soil fertility and micro-climate regulation, support agriculture and buffer natural hazards [5]. Forested ecosystems also regulate ecological processes such as water flows and carbon sequestration, with well-being benefits to people who live beyond the location of the forests [6]. Some studies, building on land multifunctionality and sustainable management, suggest integrated approaches to adaptation, for example, climate-smart agriculture for food systems [7], sustainable forest management [8], landscape approaches to land-use planning [9,10], and nature-based solutions in environmental policies [11].

Ecosystems can help people achieve multiple development objectives simultaneously, including adaptation to climate change and other hazards, but the contribution ecosystems can make depends on how lands are managed and benefits are shared [12]. Land uses are defined as the sum of management arrangements, activities, and inputs that people undertake in a certain land cover type [13]. Land uses shape ecosystem characteristics and the bundles of ecosystem services as well as any trade-offs between services over space and time [14,15]. Land-use changes often enhance the supply of one or more ecosystem services of interest at the expense of others, for example, the increase of food production may degrade regulating services [15,16]. In addition, a land-use change that is adaptive for some individuals or groups may have unintended off-site effects for others at different scales [17]. Therefore, as trade-offs create winners and losers in how people benefit from ecosystems, so land-use changes may reduce livelihood risks for some stakeholders (especially those deciding on land-use changes) but increase risks for others, locally or further afield [7,18,19].

Despite the importance of trade-offs and off-site effects in relation to making ecosystem service assessments useful and operational [20,21], there has been limited research on how land-use changes lead to trade-offs between ecosystem services (reviewed in [22]), particularly across spatial scales and beneficiaries [23,24]. Another challenge is to better understand the processes that change dominant social-ecological structures, e.g. societal learning feedback loops that can transform institutions or practices related to the management of agricultural and forest ecosystems [25,26]. In this study, we analyze how rural communities in two tropical forested landscapes in Indonesia have changed land uses to maintain their livelihoods and adapt to several environmental, economic, and social risks. We describe the impacts of major land-use changes on the supply of ecosystem services, with consequences for well-being at local (provision of products), regional (water regulation), and global scales (carbon sequestration), as well as across multiple scales (biodiversity, which supports all ecosystem services). We discuss how local land-use changes are reinforced and spread at the landscape scale and how local land-use changes can trigger larger-scale transformations to more resilient development pathways.

## Methods

### Analytical framework

**Fig 1:**
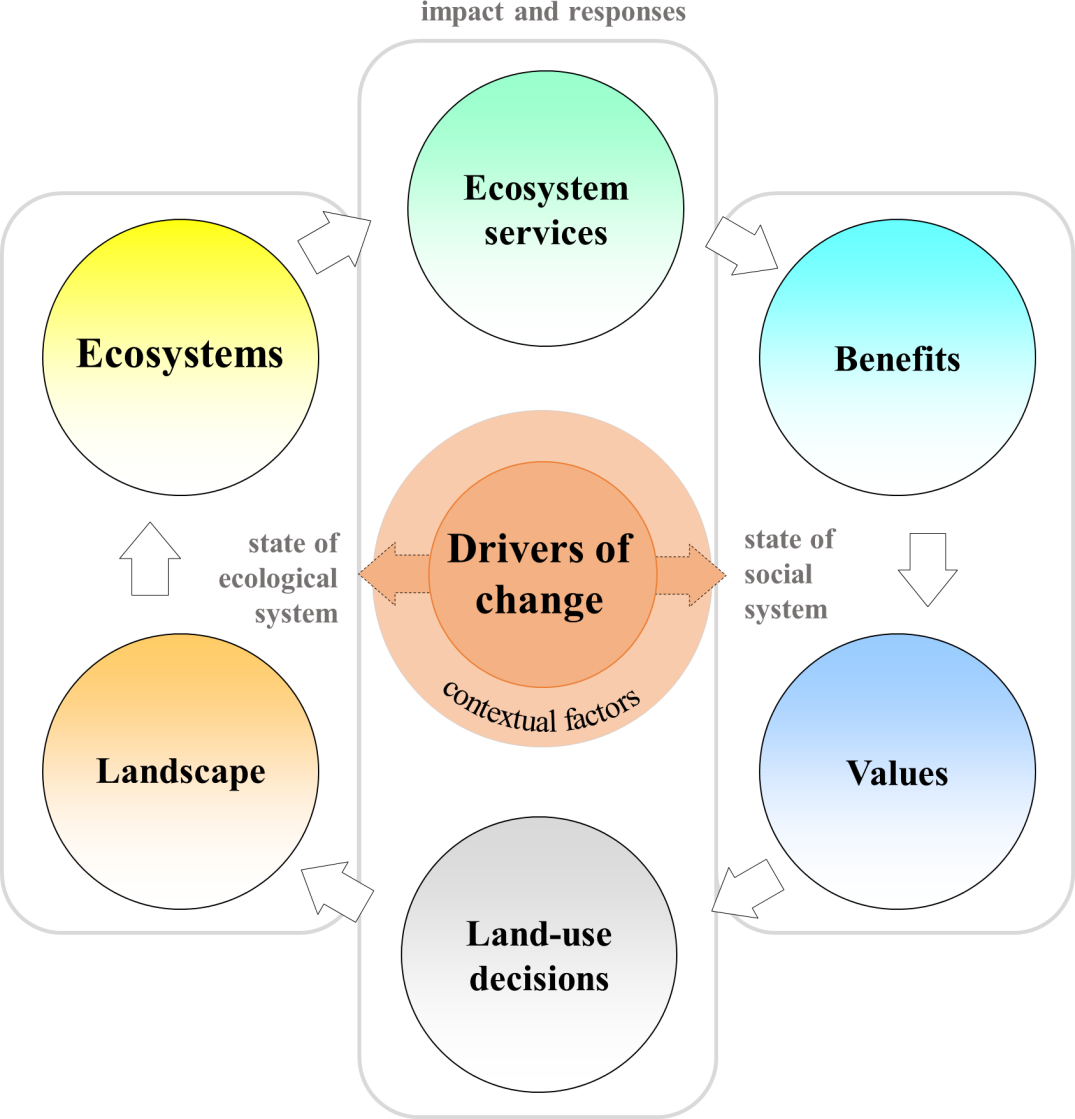
The modified ecosystem services cascade framework. Drivers of change affect the state of social and ecological systems. Changes in landscape properties or societal values influence land-use decisions and the supply of ecosystem services. A change at one point in the system triggers further changes because of the reinforcing loop of ecosystem services flows, whereby benefits derived from particular land uses lead to more changes by local people to those land-uses to ensure supply of more services.

In order to understand how land-use changes affect interactions within social-ecological systems, we used a modification of the ecosystem services cascade of Haines-Young and Potschin [27]. This framework details steps in the flow of services from ecosystems to societies: each is step mediated by decisions that determine the flow of services and benefits [28]. In our analytical framework, drivers of change affect the state of ecosystems and social systems, which in turn alter land management and the supply of ecosystem services (Fig 1). For example, frequent wildfire (as a driver of change) might convert savannah woodland to grassland (as an impact on the state of the ecosystem) and local people might decide to leave (as an impact on the social system). To reduce impacts, people can adapt by adjusting land uses (e.g. abandon agricultural fields, plant fire-tolerant trees, introduce grazing and prescribed burning), building infrastructure (e.g. create firebreaks, establish early warning systems, install new water pumps), or changing social-economic behaviors (e.g. increase awareness, organize fire-fighting groups, subscribe to insurance).

The framework highlights how land-use decisions by local actors can spread through reinforcing loops via the ecosystem service flow (circular arrows in Fig 1), which connects societal demand for ecosystem services with their supply by ecosystems. When people value socio-cultural, ecological, or economic benefits from certain land uses, they are more likely to make decisions that favor such land uses. Once implemented, these land-use decisions increase the supply of ecosystem services, which in turn increase benefits and the appreciation of the value of ecosystem services by beneficiaries who push for replication and spread of the land-use decisions that result in the supply of those services. Therefore, a reinforcing loop is created that sustains the direction of change and contributes to spreading the land use to new places and people by scaling out and up. In this way, a local change can become widespread in a landscape or region and have impacts for people far beyond the local scale. Reinforcing loops are not the only influences on the spread of land-use decisions; contextual factors include rights of access and use, livelihood priorities, and peoples’ capacities that control the human inputs necessary to co-produce ecosystem services [29].

### Study sites

Indonesia is particularly prone to natural hazards [30]: it is in a region of archipelagos vulnerable to tropical storms and volcanic activity and a large number of its people depend on natural resources-based livelihoods, such as farming, forestry, and fisheries, that are sensitive to natural hazards. Although Indonesia is rich in tropical forests, extensive areas have been lost in recent decades due to expansion of agriculture, oil palm and other tree plantations, and mining [8].

**Fig 2:**
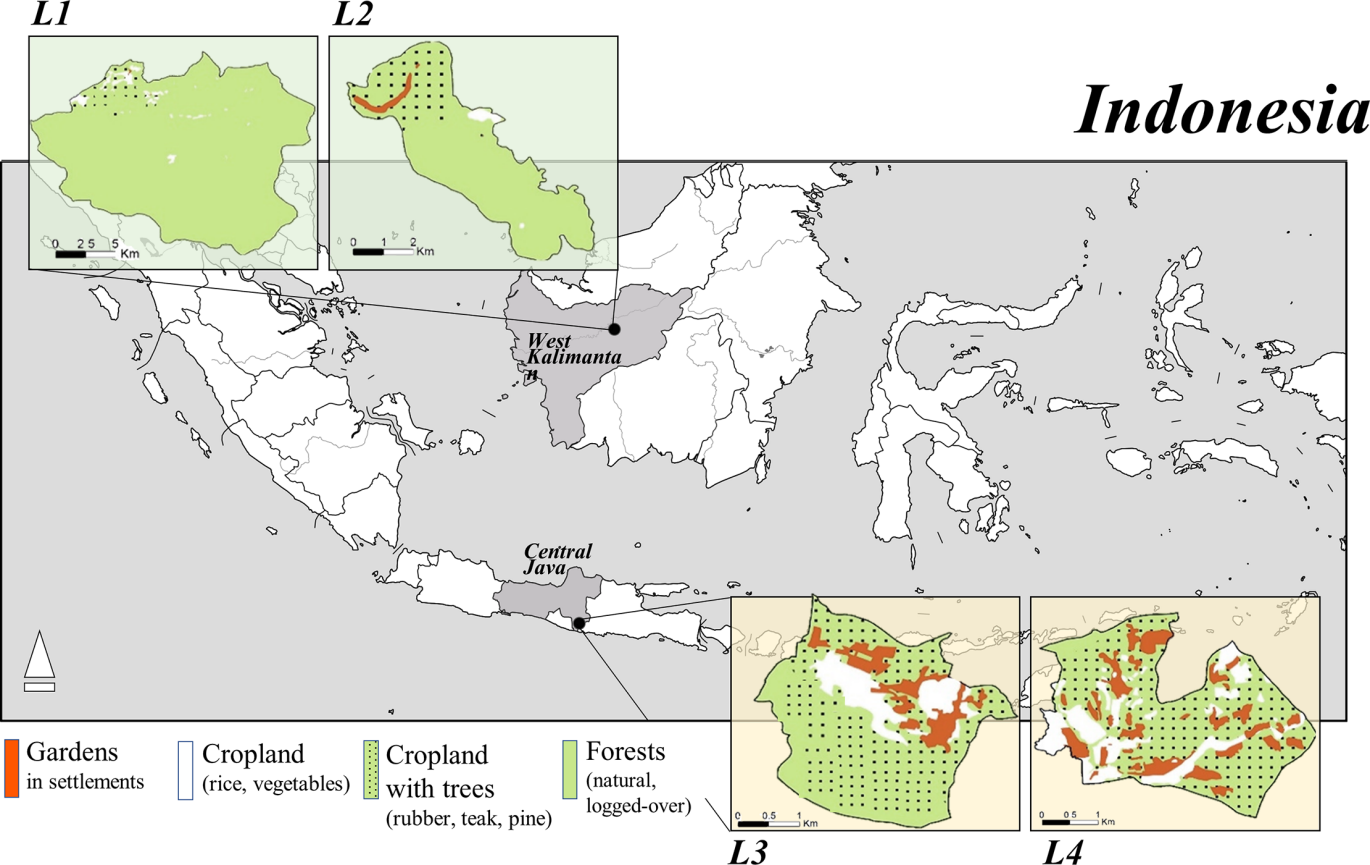
Map of the study sites. Land cover in the studied landscapes (L) in the Indonesian provinces of West Kalimantan (L1, L2) and Central Java (L3, L4) in 2014.

We selected the provinces of West Kalimantan and Central Java because of the diversity of forest cover and of drivers of change and development. Most areas of these provinces face medium to high climatic risks because of the high magnitude of natural hazards and low capacities to respond, according to the Indonesian National Board for Disaster Management [31]. Natural hazards and other sources of vulnerabilities can drive adaptation strategies among the people affected, including decisions to change land use, that favor the provision of certain ecosystem services at the expense of others. In each province, we selected two study sites in landscapes with varying forest cover (Fig 2). The sites in West Kalimantan were dominated by relatively abundant “natural” dipterocarp forests with some rubber plantations, whereas in Central Java the sites were strongly influenced by human activities, consisting of mixed cropping (rice, soya, maize) and secondary forests (mostly plantation teak and pine).

### Methodological approach

We used a transdisciplinary approach to identify how local people changed land uses to adapt their livelihoods to multiple risks, and to assess the impact of these changes on ecosystem services. The field work was conducted over the course of three rounds per province for approximately one month each between March 2014 and June 2015. In focus group discussions (20 in total), we identified the drivers of change that impacted peoples’ lives and the responses to these changes since 1994. Among the adaptation strategies reported by communities, we selected those that led to changes in land uses applied by most people over a large part of the landscape. Rural communities and their leaders who participated in this study agreed by oral consent. The field work was permitted by the pertinent Indonesia authorities and approved by CIFOR Ethics Review Committee.

We assessed the impact of land-use changes on multiple benefits provided by ecosystems in order to understand the effects on adaptation and well-being of people at different scales. Ecosystem services can support peoples’ adaptations by reducing impacts of climate hazards or other risks and strengthening capacities to respond [2,5]. In particular, provisioning services of forests and agroecosystems can help diversify local livelihoods and income sources [32]. Regulating services of water flow and purification buffer water quality and quantities with local and regional benefits [6]. In addition, global climate regulation through carbon sequestration, help avoid further climate change and represents a long-term strategy, lessening the need for adaptation [33]. Finally, biodiversity underpins many ecosystem services with benefits from local to global scales [34]; thus increasing species diversity has positive effects for timber and water supply, pest control, and regulation of soil, water and climate [35,36].

We used four indicators to assess selected ecosystem services and their evolution with land-use changes (Table 1): values of harvested products, peoples’ satisfaction with clean water availability, amount of carbon stocked in aboveground biomass, and tree species richness. Data on these indicators were collected in structured interviews (160 people and key informants) and forest inventories (120 plots). We compared the indicators for current land uses (2014) with estimations of land uses before change (1994 or 2004), assessed using Landsat 7 ETM+ images for 1994, 2004, and 2014 (US Geological Survey database path/raw: 119/60 for West Kalimantan and 119/66 for Central Java). The interpretation of satellite images was complemented by participatory mapping and ground-truthing. For estimating the amounts of biodiversity, carbon, and harvested products of past land uses, we used space-for-time substitution with analogue land uses currently found in the landscapes [37]. For clean water availability, we asked people directly about their perceptions.

**Table 1:** Overview of the indicators and methods used to assess land-use changes and their impact on ecosystem services.

Indicator	Unit	Description	Data source(s)
Land-use type	Qualitative and ha	Type and area of each land use in the village territory in 1994, 2004, and 2014.	• Remote sensing (Landsat 7 ETM) • Participatory mapping
Carbon	t C/ha	Mean aboveground carbon stocks per land-use type.	• Tree inventories
Biodiversity	Number of species	Mean tree species richness per land-use type.	• Tree inventories
Water	1 (low) - 5 (high)	Stated satisfaction (low–high) of local people with clean water availability (quantity and quality) for 1994, 2004, and 2014.	• Key informant structured interviews
Products	USD/ha/y	Estimated economic value of harvested forest and agricultural products per land-use type (i.e. actual land use for cash or subsistence per year).	• Key informant structured interviews • Secondary literature

#### Focus group discussion: Major land use changes

In focus group discussions, we identified why and how local people have adjusted land uses to respond to drivers of change since 1994. To guide discussions, we used rural appraisal techniques of participatory mapping, historical timelines, seasonal calendars, and problem-trees exercises [38,39]. For each discussion (5 per village, 20 in total), we invited 12–15 participants representing different livelihoods (farmers, forest users, off-farm workers, and local authorities), genders, and locations within the study sites. The focus group discussions lasted around 2.5 hours each and were held between March 2014 and June 2015.

#### Interviews and secondary literature: Clean water and products from the land

To assess clean water, due to lack of historical hydrological data, we asked 40 local adults per village to score their satisfaction with current and past availability of clean water, i.e. quality and quantity for domestic and agricultural purposes. They scored water conditions on a 5-point scale (from very unsatisfied to very satisfied), for the current situation, 10 years ago, and 20 years ago (i.e. 2014, 2004, 1994). Scores were drawn on a graph and the trends discussed with the interviewees. Their explanations helped us check the reasons for changes in water conditions (e.g. land-use changes, technological improvements, climate variations). People assessed the water benefits at landscape scale rather than between land uses. However, land-use changes were widespread, so we assumed they influenced perceived trends in clean water availability.

To estimate the value of harvested products from each land use, we asked key informants about harvesting frequencies, quantities, and local market prices. Crop yields per hectare per year were taken from official provincial statistics [40]. For forest products, we used harvestable tree stocks per hectare from our forest inventories, checked against tree stocks and yearly livelihood incomes from forestry from other studies in the same villages, sub-district or district (S3 Table). We then calculated average gross local monetary value of harvested products from each land-use type per hectare per year, including cash and cash-equivalents in the case of subsistence farming, i.e. actual use values without labor costs.

#### Forest inventories: Aboveground carbon stocks and diversity of tree species

Carbon stocks in aboveground biomass and tree species richness were assessed using field inventories and their mean values were used for each land-use type. We inventoried 81 plots selected using stratified random sampling based on the land-use types previously identified by remote sensing and participatory mapping (S5 Table). Sample size was defined depending on expected carbon stocks in each land-use type according to the formula suggested by Winrock International [41], and was adjusted to have at least four plots per land-use type. In circular nested plots with an area of 400 m^2^, we measured tree diameters (>2 cm) at breast height (DBH at around 1.3 m height), estimated their height, and identified species with the help of parataxonomists and databases of previous studies in the region [42].

Carbon stocks were calculated using the improved allometric equation for tropical trees [43]. Dry wood specific density data were obtained from the ICRAF Wood Density Database [44] according to the lowest level of botanical identification possible; otherwise, mean values were used. For crop land, we assumed an aboveground carbon stock of 2 t C/ha, with little likelihood of temporal change because of annual cropping and replanting [45].

## Results

### Drivers of change and response strategies

At all four study sites, livelihoods were mostly based on land-use activities. In West Kalimantan (L1-2), most people interviewed harvested rubber (90%), practiced traditional gold mining (50%), and cultivated rice for subsistence (30%). They cited extreme fluctuations in rainfall, leading to floods or drought among the drivers of change that had the most severe impact on livelihoods in the last 20 years (S1 Table). These hazards disrupted river and road transport, preventing logging and mining, with floods damaging houses, and crops, and fish ponds. The people interviewed in Central Java (L3-4) were smallholders who cultivated rice and vegetables (100%), and sometimes also raised goats and cows (60%). They identified wildlife grazing, drought, and pest outbreaks as major hazards, impacting livelihoods by reducing agricultural production (by up to half), clean water availability, and indirectly decreased farm labor and increased food prices.

A range of adaptations to maintain livelihoods were evident in the study population. In West Kalimantan, interviewees repaired flood-damaged houses, fields and fishponds or relocated them, harvested forest products such as fruit, birds, and deer, or borrowed money. In Central Java, they bought water and food, worked off-farm, temporarily migrated to cities for jobs, sold livestock or plantation timber, and changed diets (eating less rice, feeding animals with leaves). In both provinces, farmers changed crop varieties, reduced harvest times, and used fertilizers and pesticides. Other technical adaptations included building irrigation channels and wells, pumping or transporting water, stabilizing slopes with terracing (Central Java) or protecting vegetation (West Kalimantan).

### Major land use changes

Local people reduced livelihood risks through land-use changes (Table 2, L1-4). In West Kalimantan, people converted forests to rubber plantations to diversify livelihoods and maintain their income in case of floods and droughts (Table 3, L1). The area of rubber plantations, and the number of people working them, have increased by around 40% in the last 20 years, according to the participants in the mapping exercise in L1. Farmers reported that since the 1990s, they have expanded the traditional practice of shifting cultivation, whereby forests are cut and burned to grow upland rice. After a few years of rice cultivation, they replace the rice with rubber trees. According to the farmers, rubber plantations offer a flexible alternative to cultivation and a supplementary income source because their productivity is less affected by drought than is cropping. In addition, rubber trees can be tapped at any time and the harvested latex stored, allowing farmers to wait for good times to sell (prompted by urgent need or high prices).

**Table 2:** Description of the major land-use changes (L1–4) that the local people undertook to adapt and maintain well-being under the impacts of drivers of change (source: Focus group discussions).

Study site	Landscape intervention	Land-use changes and actors	Description of specific land management measures
L1	Forest conversion	Farmers convert logged-over forests to rubber plantations	- clear-cut forests through slash and burn - maintain or plant fruit trees (e.g. durian, rambutan) - cultivate rain-fed rice (2–3 y) and plant rubber trees (~30 y) - fertilize, remove competing vegetation, tap rubber
L2	Forest protection	Village leader introduces deforestation ban to protect forests	- introduce rule to ban deforestation in less degraded forests - harvest NTFPs and trees for local uses (selective logging) - do not cut down big trees and fruit trees along rivers
L3	Agroforestry	Villagers plant trees in gardens (forest gardens)	- plant teak in gardens coordinated by farmer association - assist natural regeneration, thin and prune trees, fertilize - follow rules for harvest (DBH>20 cm, age>20 y), replant (1:10)
L4	Reforestation	Farmers reforest less productive croplands	- abandon less productive croplands on slopes - plant or assist regeneration of teak and mahogany - follow social norms to replant trees after cutting

**Table 3:** Objectives that triggered land-use changes, enabling factors and perceived effects (source: Focus group discussions).

Study site	Objective of land-use changes	Contextual factors enabling land-use changes	Perceived effects of land-use changes
L1	Increase income opportunities despite extreme weather	- good rubber prices - new settlement, bridge, road - government inputs for rubber (seedlings, techniques, fertilizers)	- more flexible and diversified livelihoods - less clean water in rivers (for fishing, drinking, washing) - more severe floods when heavy rain
L2	Maintain (scarce) natural resources for future local needs	- political change (new village and leader) - experiences with forest changes (logging, mining, shifting cultivation) - perceived increasing impact of climate variability (drought, floods, heat)	- more efficient use of degraded land - little improvement in clean water (but more expected)
L3	Diversify income opportunities	- coordination by farmer association - support from NGOs - experiences with water shortages - good teak demand and prices	- more flexible and diversified livelihoods - more water in dry season for cultivation
L4	Maintain (low) land productivity (droughts, wildlife, pests)	- low soil fertility (far from river, rocky, slopes) - lack of labor (migration and aging)	- fewer harvest losses from drought, pests, and wildlife than for crops - lower workload than for crops

Another change was the introduction of a new village rule to preserve forests in 2011 (Table 2, L2), which banned shifting cultivation in less degraded forests, mostly on hills (around 45% of the village territory). In these forests, people could harvest non-timber forest products (NTFPs) such as firewood, rattan, agarwood, and birds, or selectively log a few trees for local use, but not along rivers. The village chief explained that the rule was established to “*avoid that our next generations experience difficulties in finding natural and forest resources and face intense floods and hot weather*”.

In Central Java, a new land use involved planting trees on private lands near settlements which helped diversify farmers’ livelihoods and income opportunities (Table 3, L3). In the focus group discussions, the villagers reported that in the late 1980s, a farmer started this agroforestry practice in his garden and some years later it was replicated by neighbors who created forest gardens (around 60% of gardens). The farmers formed an association to coordinate management practices that was later supported by an NGO. In 2004, the forest gardens of three hamlets became a certified community forest and they were given a sustainable natural resource management label called *Lembaga Ekolabel Indonesia* (LEI 2004 village certification book). According to the head of the certified forests group in L3: “*at the beginning we planted trees to complement income from crops*, *but later on we also realized the positive impact on water springs*”.

Another widespread land-use change in Central Java was the abandonment of less productive croplands of rice, soya, and peanut (around 15% of all rain-fed cropland). Although some rice fields are close to the river and cultivated up to three times per year, most are on rain-fed terraced slopes. Farmers reported that these less productive croplands were cultivated with rice only if enough rain was expected and were otherwise planted with other crops or left fallow. However, due to rainfall variability, harvest failures were frequent. Farmers reforested some less productive fields by allowing natural regeneration to occur or planting teak and mahogany (Table 2, L4). This land-use change spread during the early 2000s, when farmers reported more frequent harvest losses because of foraging by monkeys and wild boars at the village margins.

### Biodiversity

**Fig 3:**
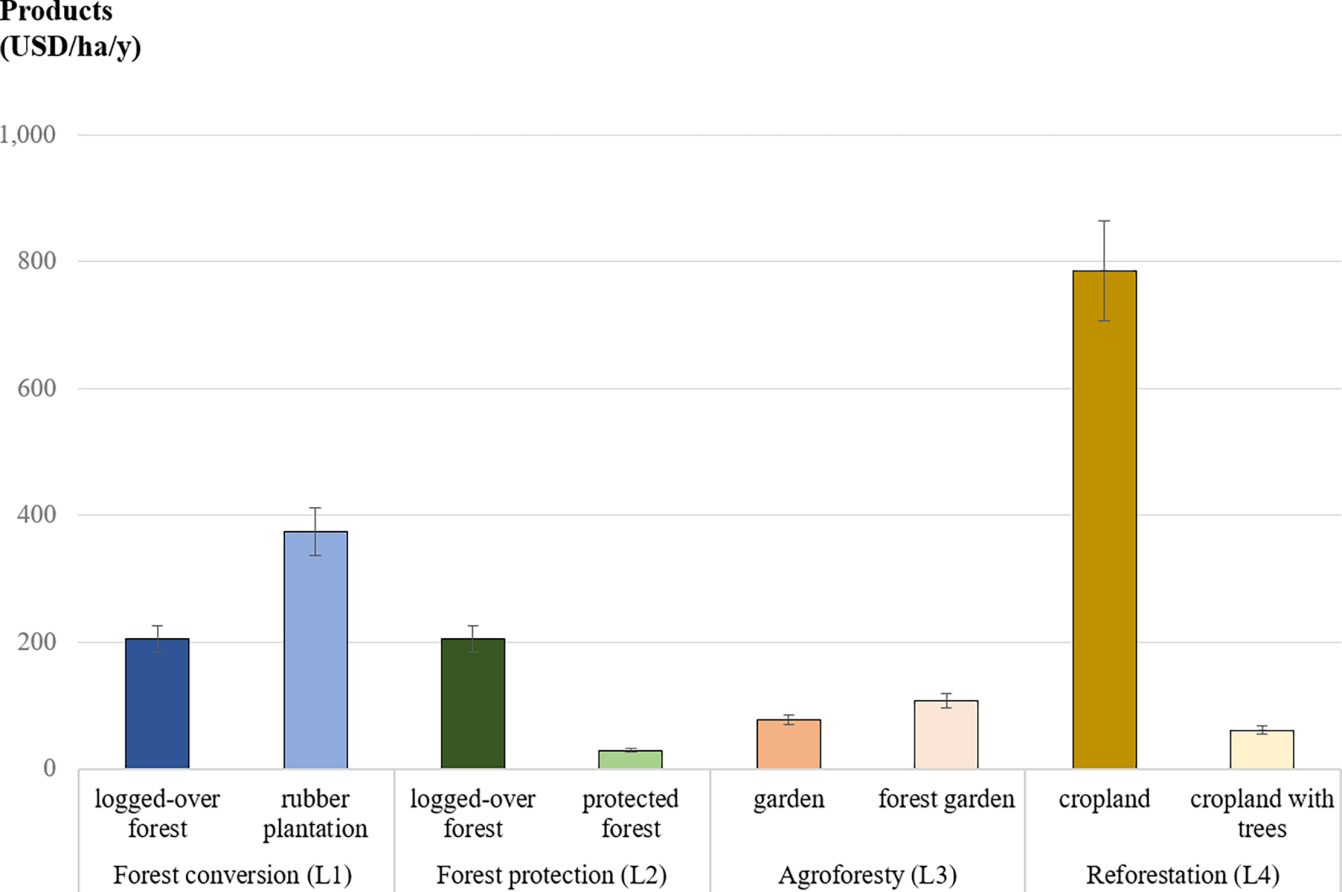
Mean number of tree species per land use (± SD) that were changed by local people as part of their adaptation strategies to hazards (L1–4).

Logged-over or protected forests in West Kalimantan hosted similar tree species richness (mostly *Shorea* spp., *Syzygium* spp., and *Turpinia* spp.) (Fig 3, S2 Table). A few rubber plantations (*Havea braziliensis*) were mixed with fruit trees, such as mango or durian, which led to an average of around 3 species in this land use. In Central Java, croplands and gardens had low tree species richness (0–3). Gardens, mostly planted with cassava, maize, and medicinal herbs, were sometimes mixed with coconut, banana, and bamboo trees. The tree species richness was higher (up to 5) in forest gardens and mostly included teak (*Tectona grandis*) or mahogany (*Swietenia macrophylla*). The same species were used to reforest less productive croplands in addition to some natural regeneration with shrubs and other trees such as *Acacia* spp. and *Pterocarpus* spp.

### Products from the land

**Fig 4:**
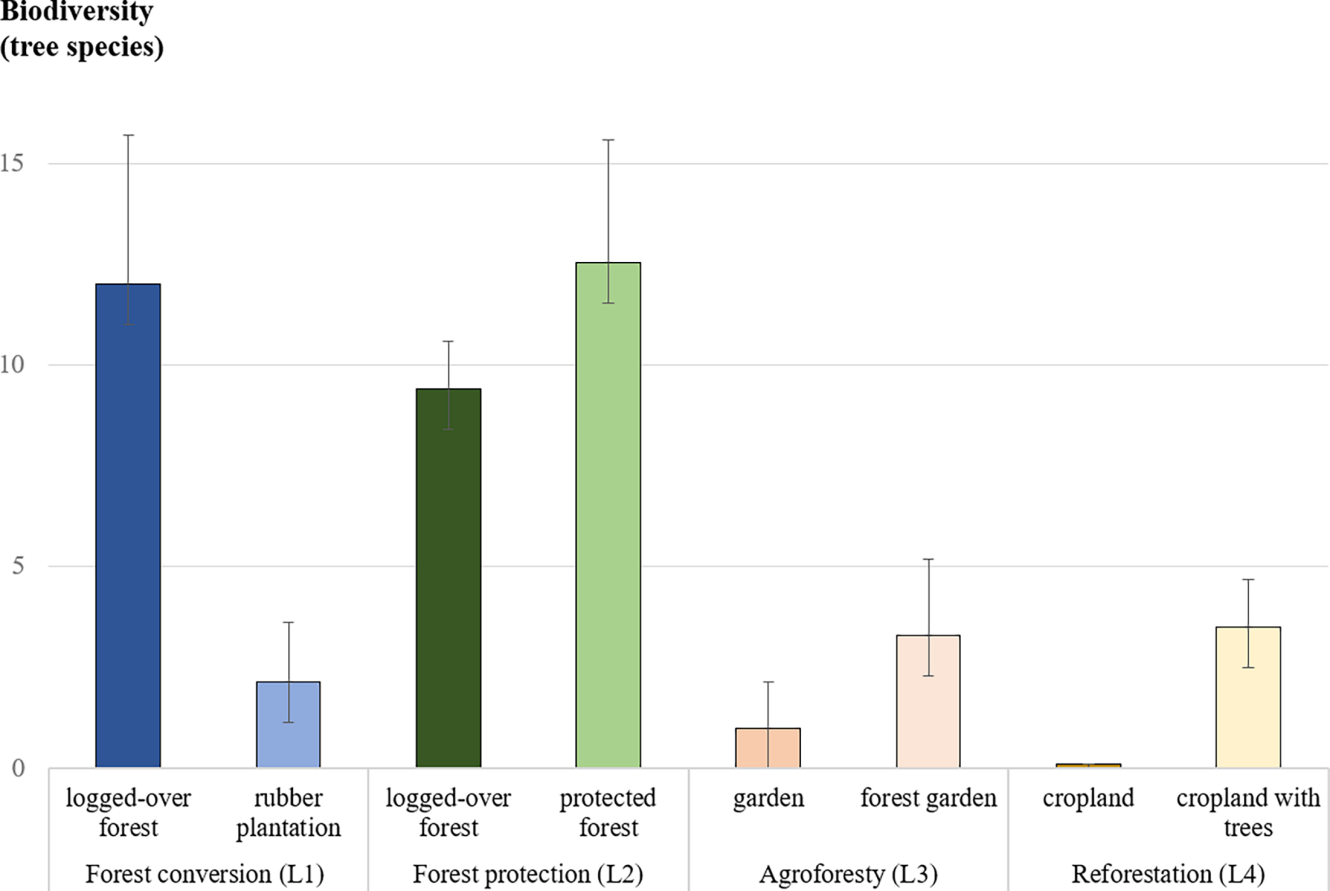
Mean values of harvested products from the land (USD/ha/y ± 10% uncertainties). Land uses that were changed by local people as part of their adaptation strategies to hazards (L1–4).

In West Kalimantan, local livelihoods depended on several forest and tree products such as timber, rubber, and other NTFPs (e.g. agarwood, fruits, deer, birds). The main timber species harvested for building or trade was the Bornean ironwood (*Eusideroxylon zwageri*); however, it was becoming increasingly rare according to the forest users in the focus group discussions (L1-2). People extracted timber for an estimated value of 180 USD/ha/y and collected NTFPs worth 30 USD/ha/y (Fig 4). Rubber plantations were the most profitable land use, whose latex collection was worth 375 USD/ha/y.

In Central Java, the highest income source was croplands planted with rice in irrigated fields near the river and harvested up to three times per year, or in rain-fed fields and harvested once a year (785 USD/ha/y). During the first planting season, farmers explained that they preferred to cultivate the rain-fed fields with red rice (an early-maturing drought-resistant variety). When the least productive croplands were abandoned and trees planted, the harvested product value fell to 40 USD/ha/y. Food (vegetables and cassava) and medicinal plants in gardens were worth 80 USD/ha/y, and when mixed in with agroforestry in forest gardens, reached 110 USD/ha/y.

### Clean water

**Fig 5:**
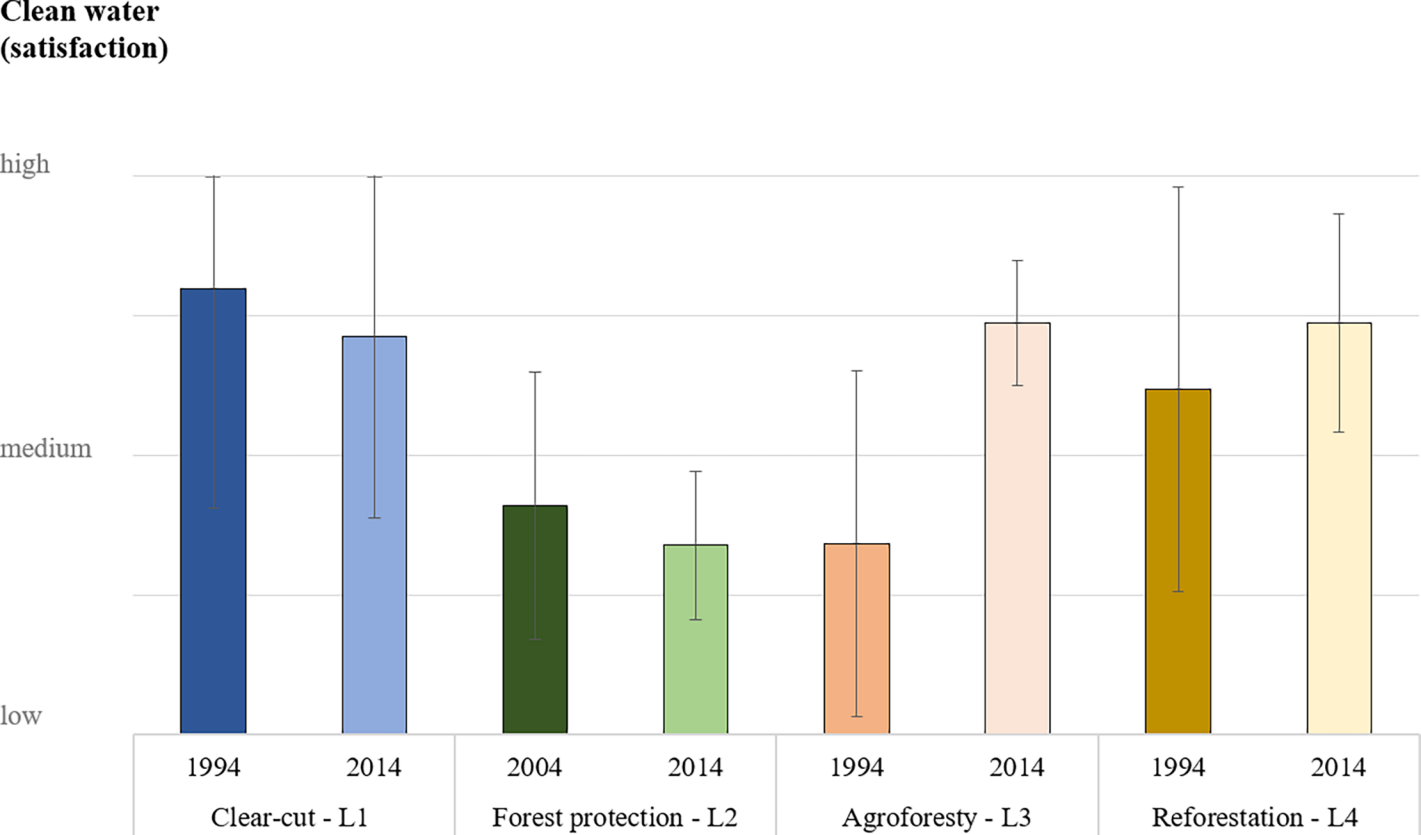
Local peoples’ scores of clean water availability during the last 10–20 years (from high to low satisfaction ± SD). Changes in satisfaction with clean water availability during the periods when the selected major land-use changes occurred as part of the adaptation strategies to hazards (L1–4).

The perception by local people of clean water availability evolved differently in the two regions over the last 20 years (Fig 5). In West Kalimantan, clean water availability decreased slightly at places where forests were converted into rubber plantations (L1) or protected (L2). However, the time elapsed since forest protection started at L2 may be too short to have noticeable effects on water. In Central Java, clean water perceptions have improved, during the last 20 years, when the number of trees increased in the landscape (L3–4). Several respondents connected these trends with recent changes in forests, such as the building of new wells or water channels (S4 Table). For example, villagers in West Kalimantan reported that “*shifting cultivations and gold mining activities are decreasing the soil fertility and water quality*” (L1) or “*in the future the water might get better because of the new regulations that prevent the mining*” (L2). In Central Java, interviewees mentioned that *“water conditions are improving because the community forest grows very well”* (L3) and that “*there are many reforestation activities that if they continue will help us to have more secure sources of fresh water”* (L4).

### Carbon

**Fig 6:**
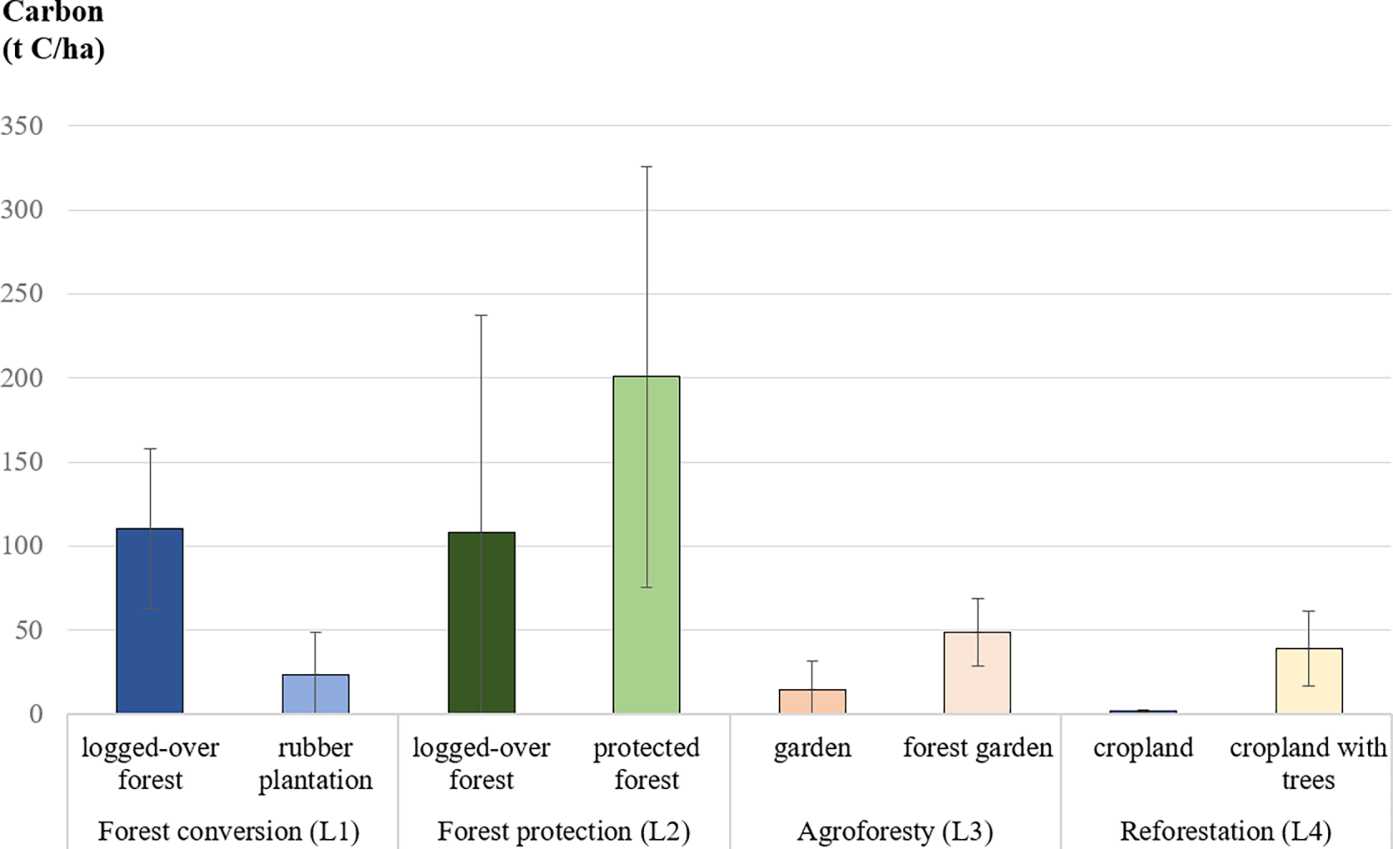
Mean carbon stock (t C/ha ± SD) in aboveground biomass. Measurements were taken for the land uses that were changed by local people as part of their adaptation strategies to hazards (L1–4).

Carbon stocks in aboveground biomass were highest in the semi-natural protected forests (198 t C/ha) and in old logged-over forests (130 t C/ha) in West Kalimantan (Fig 6 and S5 Table). In rubber plantations, carbon stocks were 80% less than in logged-over forests (L1). In Central Java, gardens and croplands had the lowest aboveground carbon stocks (15 t C/ha and 2 t C/ha, respectively). Trees planted in these lands stored up to 49 t C/ha (L3–4).

### Trade-offs

**Fig 7:**
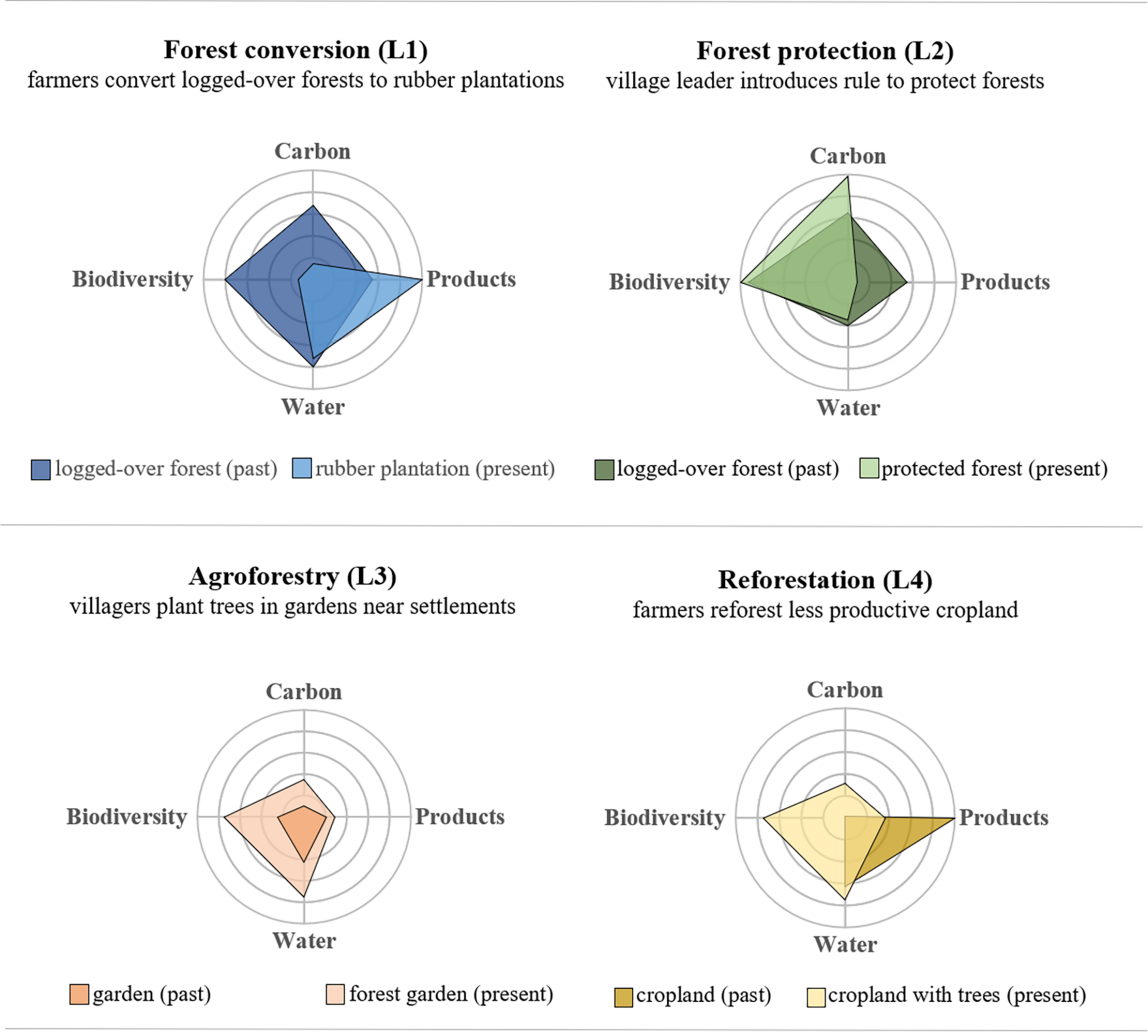
Changes in ecosystem services (land products, carbon sequestration, water purification and regulation) and biodiversity before and after selected land-use changes as per people’s adaptation strategies. The value of each indicator is normalized from 0 (minimum possible value at the center of the spider plot) to 5 (maximum observed value on the outermost circle).

Selected land-use changes increased most ecosystem services, but some trade-offs occurred (Fig 7). In West Kalimantan (L1), the conversion of logged-over forests into rubber plantations favored products at the expense of biodiversity, carbon, and water benefits. Conversely, in Central Java (L4), the reforestation of less productive cropland resulted in a decrease of products and an increase of biodiversity, carbon, and clean water. Forest protection in West Kalimantan (L2), increased biodiversity and carbon stocks but limited the income from forest products. The agroforestry practices in forest gardens in Central Java (L3) increased all ecosystem services without any particular trade-offs between biodiversity, carbon, products, and clean water.

## Discussion

### Drivers and impacts of land use change

Over the last 20 years, local people in the study sites have changed land uses by adjusting their management of trees to reduce risks to livelihood linked to natural resource scarcity, low agricultural productivity, and climate hazards. Similar to the Indonesia cases, other rural communities in tropical landscapes have started local initiatives to manage forested areas to improve ecosystem services and adaptation benefits. For example, farmers in Southeast Asia adapted land uses by mixing plantations of rubber, coffee, or cacao with crops in agroforestry systems [46,47]. Farmers in the Sahel reforested dry lands to make livelihoods resilient to drought following changes in governance and farming practices [48]. Smallholders in the Ecuadorian Andes planted trees on agricultural lands or protected forests to prevent burning and cattle grazing and to increase economic diversification [49], motivated by the community perception that forest conversion to other uses would negatively affect water quality and availability.

Changes in land uses to increase local benefits from ecosystems have consequences for other services that span spatial scales. In the Indonesia case studies, local people modified forest and agroecosystems to change supply of products, diversify livelihoods and reduce risks, but these changes impacted regulating services that benefit people in other areas. Land-use decisions that increased provisioning services for local benefits, like in the case of conversion of forests to rubber plantation, led to trade-offs with regulating services that were then reduced, providing fewer benefits at larger scales. Conversely, restoration of regulating services for water by reforesting cropland or protecting forest patches led to a decrease in local benefits from provisioning services). Similar trade-offs or synergies between provisioning and regulating services have been reported [12,15], as well as among regulating services [50,51].

Local strategies for adaptation based on land-use changes result in co-benefits and trade-offs at the global scale. Three of four land-use strategies (L2–4) increased local and regional benefits (more products and cleaner water), but also global benefits for climate mitigation (more carbon stocks). Such strategies met the converging interests of local and global stakeholders for solutions to climate change. However, local strategies can also result in trade-offs for carbon sequestration, as for conversion of forests to rubber plantations (L1), where interests of local people to strengthen livelihoods diverged from the global priority to reduce carbon emissions. Understanding the impact of local adaptation strategies on ecosystem services that can have benefits at the global scale, can help implement successful actions for climate change that account for different stakeholders’ interests. International policy initiatives on climate change mitigation (e.g. REDD+, climate-smart agriculture) that consider local ecosystem benefits are more likely to be legitimate and long-lasting [52–54]. At the same time, such initiatives should be aware of local adaptation strategies that might affect forests and carbon permanence.

### Mechanisms reinforcing decisions to change land use

When local actors perceive that strategies based on small-scale land use changes are successful, they can expand strategies and spread change at landscape scale. As our analysis showed, a single-farmer initiative or a rule made by a village chief may be followed by others. The ecosystem services framework helps highlight how perceived increases in certain benefits from ecosystems can be reinforced through land management decisions. Attempting large-scale change without preliminary small-scale change is difficult and risky [55]. However, even if changes spread within a community, disparities may exist between groups due to varying power relations, capacities, dependencies, or access rights that should be considered [56,57]. Positive effects on ecosystem services that are socially accepted and inclusive create feedback loops that shape trajectories of social–ecological systems [58,59].

Perception by local actors that land-use changes improve livelihoods and reduce risks creates a reinforcing loop that increases the spread of such changes: supply of more ecosystem services leads to broader adoption of change in land-use (Fig 1). Several people at the study sites appreciated new land uses that offered more flexible, diverse, and resilient income opportunities (e.g. rubber in L1 or teak in L3–4). They also valued improved clean water conditions as co-benefits of the land-use changes (due to tree cover in L2–4). As represented in the conceptual framework, the ecosystem service flows connect supply from ecosystem with demand of people who can decide to influence these flows. When more people appreciate certain ecosystems or landscape states, so more decisions are implemented that shape landscape characteristics according to peoples’ interests. Since changes in societal values due to observed outcomes of land-use decisions can trigger reinforcing loops, it is important that people have opportunities to explore different strategies and learn from experience [60,61]. This outcome may be achieved by empowering local groups to develop and implement new land-uses and practices.

### Factors facilitating decisions to change land uses

The introduction of new land uses by local actors to improve livelihoods and reduce risks is facilitated by the states of or changes in social or ecological systems, which create “windows of opportunity” [62,63]. As shown by the response of the communities studied in West Kalimantan, floods, drought, or natural resource scarcity can trigger changes in forest use. It has been reported that extreme weather variability and restricted forest access due to logging concessions have triggered adjustments in land management in other areas in the region [64,65]. Other opportunities for new land uses can be triggered by changes in the social–institutional context at different scales; for example, when a new local leader introduces rules for use and management of community forests like in the case of West Kalimantan. In addition, external factors that trigger changes in land-use decisions include new forest and climate policies, demographic change, or economic development. Changes in government forest policies and in levels of control were common in the colonial and reformation period in Java and determined the land-use decisions made by local people e.g. to plant or cut trees [66,67]. The analysis in the villages in Central Java showed that also a lack of labor due to migration and aging populations can lead to reforestation of abandoned agricultural land. In addition, increased commodity prices or construction of new roads or water systems also influenced peoples’ uses of ecosystems.

Land-use changes that lead to improved livelihoods may not spread automatically because reinforcing loops that can promote certain ecosystem services depend on human actions and contextual factors. Dominant rules and power relations, values, and knowledge can hinder or facilitate people’s adaptation decisions and actions [68–70]. These factors influence people’s decisions with repercussions on the flows of ecosystem services. As shown in the case studies, the experiences of farmers affected by logging or water shortages, as well as knowledge of market prices and the values and rules developed through community organizations, facilitated changes in land uses. Other factors might hinder change, such as lack of land tenure rights and infrastructure (including market access). Overall, contextual factors influence land-use decisions and other inputs to co-production and delivery of ecosystem services to final beneficiaries [71] and can change the trajectory of social–ecological systems [72,73].

### Implications of local land-use decisions at larger scales

Local adaptation strategies can introduce novel ways of managing ecosystems that then spread at the landscape scale through reinforcing loops in the ecosystem services flow and have impact beyond the local scale. Such responses have been described as transformative adaptations [2,74,75], although a consensus on their definition is lacking [55] (but see [76]). In contrast, coping responses are usually reactive, tactical, and short-term [77] and incremental adaptations tend to be anticipatory responses that extend current practices, but without changing prevailing systems of social organization, economic structures, and modes of production [77,78].

Transformative adaptations are generally collective strategies, undertaken at large scale or intensity, novel to the prevailing social-ecological system, and that cause major system changes [79]. In addition, they impact at several scales and challenge dominant feedback loops in the system [55]. Transformational responses might be required to address long-term, large-scale, nonlinear, and uncertain changes such as those triggered by climate change [73,80]. Strategies that seek to cope with, or incrementally adapt to, changed circumstances, may be insufficient when changes are particularly extreme or rapid, and where people are especially vulnerable [81].

Successful bottom-up land-based strategies offer the prospect of promising pathways for development that can be replicated and scaled up. Landscapes can provide multiple ecosystem services that support the livelihood needs of those managing them and provide co-benefits for people located more distantly. Whoever controls access to the land usually derives benefits from provisioning services, but people further away also benefit from regulating services via off-sites effects. Certain land-use practices are already suited to, and embedded in, local contexts, thereby increasing the chances of sustainability and success. As shown in the case studies, people may have local initiatives in place to protect or increase tree cover in the landscape.

Land-based approaches that build on local initiative with inclusive benefits and minimized trade-offs can contribute to achieving several development objectives simultaneously while having greater impact. This is particularly relevant with the increase in international initiatives on climate change, biodiversity and sustainable development such as the UN Sustainable Development Goals and the UNFCCC Paris Agreement. The design of Reduction of Emissions from Deforestation and Forest Degradation (REDD+) initiatives that consider multiple effects of adjustments in land-use strategies could leverage on local co-benefits for adaptation to help spread and scale-up new forest management practices.

## Conclusion

In this paper, we illustrated four cases of major land-use changes adopted by local people in response to multiple risks in two rural regions of Indonesia. Local people converted, protected, or planted trees in their landscapes to diversify local livelihoods and maintain land productivity under changing conditions such as climate variation and natural resource scarcity. Changes in land use mostly affected provisioning services of forests and agricultural ecosystems and produced local benefits, but also affected biodiversity and the regulating services of water quality and quantity and carbon sequestration, which have impacts beyond the local scale.

Our assessment of the impact of local land-use changes on products, water, climate, and biodiversity revealed some multiple benefits, but also trade-offs and off-site effects that were not initially considered by the people who initiated the changes; an important consideration in operationalizing ecosystem assessments. Not all land-use changes simultaneously meet multiple development and climate objectives, because actors at different scales may have diverging interests. Widespread changes in land uses entail shifts in peoples’ priorities, practices, and rules related to ecosystems and their benefits. New perceptions and strategies developed by local communities can arise from learning and experiential knowledge of the effects of change. Positive feedback loops from land-use changes with local benefits, combined with enabling contextual factors, can spread new land uses to different people and places (i.e. can scale land-use changes up and out). In this way, some land-use changes can radically modify large areas in novel ways that alter current dominant feedback loops and affect different spatial scales. Changes in social–ecological systems with such characteristics have been associated with transformative adaptations.

Ecosystem services assessments that consider feedback loops and multiple impacts on different ecosystem services and beneficiaries can help environmental managers and policy makers design and implement more locally appropriate and sustainable land-use decisions. The complexities and uncertainties of the impact of drivers of global change might require radical changes. However, such changes imply shifts in current values related to social-ecological systems can be challenging because of dominant views, traditions, and the interests of powerful stakeholders. Therefore, building on currently emerging local adaptation pathways that demonstrate multiple benefits across scales can help strengthen and scale up responses to climate change and other sources of vulnerability.

## Supporting information

S1 Table Climatic and non-climatic drivers of landscape changes in the study sites. Top 3 and other unranked drivers of change in each study site identified by local communities (L1-4).
(XLSX)

S2 Table Tree biodiversity per land use. Summary of statistical information on the sample plots and list of species per land use in each study site (L1-4).
(XLSX)

S3 Table Detailed information on land products per land use. Information on land products harvest quantities, frequency, and incomes per land use in each study site (L1-4).
(XLSX)

S4 Table Detailed information on water conditions. Summary of statistical information on people’s satisfaction with water conditions and qualitative explanations of trends in the last 20 years in each study site (L1-4).
(XLSX)

S5 Table Detailed information on carbon estimations. Summary of statistical information on carbon estimations of above ground biomass per land use in each study site (L1-4).
(XLSX)

## References

[1] Steffen W, Richardson K, Rockström J, Cornell SE, Fetzer I, Bennett EM, et al Planetary boundaries: Guiding human development on a changing planet. Science (80-). 2015;347 doi: 10.1126/science.1259855 25592418 10.1126/science.1259855

[2] Lavorel S, Colloff MJ, McIntyre S, Doherty MD, Murphy HT, Metcalfe DJ, et al Ecological mechanisms underpinning climate adaptation services. Glob Chang Biol. 2015;21: 12–31. doi: 10.1111/gcb.12689 25131443 10.1111/gcb.12689

[3] Millenium Ecosystem Assessement. Ecosystems and Human Well-Being Washington DC: Isalnd Press; 2005.

[4] Angelsen A, Jagger P, Babigumira R, Hogarth NJ, Bauch S, Börner J, et al Environmental Income and Rural Livelihoods: A Global-Comparative Analysis. World Dev. 2014;64: S12–S28. doi: 10.1016/j.worlddev.2014.03.006 10.1016/j.worlddev.2014.03.006 PMC7220182 32405139

[5] Sudmeier-Rieux K, Masundire H, Rizvi A, Rietbergern S. Ecosystems, Livelihoods and Disasters: An integrated approach to disaster risk management. Ecosystem Management Series No. 4. IUCN; 2006.

[6] Pramova E, Locatelli B, Djoudi H, Somorin O a. Forests and trees for social adaptation to climate variability and change. Wiley Interdiscip Rev Clim Chang. 2012;3: 581–596. doi: 10.1002/wcc.195

[7] Harvey CA, Chacón M, Donatti CI, Garen E, Hannah L, Andrade A, et al Climate-Smart Landscapes: Opportunities and Challenges for Integrating Adaptation and Mitigation in Tropical Agriculture. Conserv Lett. 2014;7: 77–90. doi: 10.1111/conl.12066

[8] FAO. Global Forest Resources Assessment 2015 [Internet]. Rome: UN Food and Agriculture Orgnaization; 2015 Available: www.fao.org/forestry/fra

[9] Scherr SJ, Shames S, Friedman R. From climate-smart agriculture to climate-smart landscapes. Agric Food Secur. BioMed Central; 2012;1: 12 doi: 10.1186/2048-7010-1-12

[10] Sayer J, Sunderland T, Ghazoul J, Pfund J-L, Sheil D, Meijaard E, et al Ten principles for a landscape approach to reconciling agriculture, conservation, and other competing land uses. Proc Natl Acad Sci. National Academy of Sciences; 2013;110: 8349–8356. doi: 10.1073/pnas.1210595110 23686581 10.1073/pnas.1210595110 PMC3666687

[11] European Union. Towards an EU Research and Innovation policy agenda for Nature-Based Solutions & Re-Naturing Cities [Internet]. Luxemburg: Publications Office of the European Union; 2015 doi: 10.2777/765301

[12] Bennett EM, Peterson GD, Gordon LJ. Understanding relationships among multiple ecosystem services. Ecol Lett. 2009;12: 1–11. doi: 10.1111/j.1461-0248.2009.01387.x 19845725 10.1111/j.1461-0248.2009.01387.x

[13] FAO, UNEP. The future of our land: facing the challenge. Guidel Intergrated Plan Sustain Mnanagement L Resour. 1999; 1–8. Available: www.fao.org/docrep/004/x3810e/x3810e00.htm%0A

[14] Daw T, Brown K, Rosendo S, Pomeroy R. Applying the ecosystem services concept to poverty alleviation: the need to disaggregate human well-being. Environ Conserv. 2011;38: 370–379. doi: 10.1017/S0376892911000506

[15] Rodríguez JP, Beard TDJ, Bennett EM, Cumming GS, Cork SJSJ, Agard J, et al Trade-offs across Space, Time, and Ecosystem Services. Ecol Soc. 2006;11: 28 doi: 10.2307/2390206

[16] Foley JA. Global Consequences of Land Use. Science (80-). 2005;309: 570–574. doi: 10.1126/science.1111772 16040698 10.1126/science.1111772

[17] Adger WN, Arnell NW, Tompkins EL. Adapting to climate change: perspectives across scales. Glob Environ Chang. 2005;15: 75–76. doi: 10.1016/j.gloenvcha.2005.03.001

[18] Reyers B, Biggs R, Cumming GS, Elmqvist T, Hejnowicz AP, Polasky S. Getting the measure of ecosystem services: A social-ecological approach. Frontiers in Ecology and the Environment. 2013 pp. 268–273. doi: 10.1890/120144

[19] Locatelli B, Catterall CP, Imbach P, Kumar C, Lasco R, Marín-Spiotta E, et al Tropical reforestation and climate change: Beyond carbon. Restor Ecol. 2015;23: 337–343. doi: 10.1111/rec.12209

[20] Tallis H, Polasky S. Mapping and valuing ecosystem services as an approach for conservation and natural-resource management. Ann N Y Acad Sci. 2009;1162: 265–283. doi: 10.1111/j.1749-6632.2009.04152.x 19432652 10.1111/j.1749-6632.2009.04152.x

[21] de Groot RS, Alkemade R, Braat L, Hein L, Willemen L. Challenges in integrating the concept of ecosystem services and values in landscape planning, management and decision making. Ecol Complex. 2010;7: 260–272. doi: 10.1016/j.ecocom.2009.10.006

[22] Seppelt R, Dormann CF, Eppink F V., Lautenbach S, Schmidt S. A quantitative review of ecosystem service studies: Approaches, shortcomings and the road ahead. J Appl Ecol. 2011;48: 630–636. doi: 10.1111/j.1365-2664.2010.01952.x

[23] Robards MD, Schoon ML, Meek CL, Engle NL. The importance of social drivers in the resilient provision of ecosystem services. Glob Environ Chang. Elsevier Ltd; 2011;21: 522–529. doi: 10.1016/j.gloenvcha.2010.12.004

[24] Myers SS, Patz JA. Emerging Threats to Human Health from Global Environmental Change. Annu Rev Environ Resour. Annual Reviews; 2009;34: 223–252. doi: 10.1146/annurev.environ.033108.102650

[25] Rickards L, Howden SM. Transformational adaptation: Agriculture and climate change Crop and Pasture Science. CSIRO Publishing; 2012 pp. 240–250. doi: 10.1071/CP11172

[26] Chung Tiam Fook T. Transformational processes for community-focused adaptation and social change: a synthesis. Clim Dev. Taylor & Francis; 2017;9: 5–21. doi: 10.1080/17565529.2015.1086294

[27] Haines-Young R, Potschin M. The links between biodiversity, ecosystem services and human well-being. Ecosyst Ecol A new Synth. 2010; 110–139. doi: 10.1017/CBO9780511750458.007

[28] Spangenberg JH, von Haaren C, Settele J. The ecosystem service cascade: Further developing the metaphor. Integrating societal processes to accommodate social processes and planning, and the case of bioenergy. Ecol Econ. Elsevier B.V.; 2014;104: 22–32. doi: 10.1016/j.ecolecon.2014.04.025

[29] Horcea-Milcu AI, Leventon J, Hanspach J, Fischer J. Disaggregated contributions of ecosystem services to human well-being in low-intensity farmland. Reg Environ Chang. Springer Berlin Heidelberg; 2015; 117–163. doi: 10.1007/s10113-016-0926-2

[30] EM-DAT. The CRED/OFDA International Disaster Database. In: Université Catholique de Louvain–Brussels [Internet]. 2017 [cited 10 Jan 2017]. Available: www.emdat.be

[31] BNPB—National Agency for Disaster Management. Drought Hazard in Indonesia [Internet]. 2012 [cited 11 Jan 2017] p. 1. Available: http://geospasial.bnpb.go.id/wp-content/uploads/2012/10/2012-10-16_Hazardmap_Drought_risk_assessment_2011.pdf

[32] Wunder S, Börner J, Shively G, Wyman M. Safety Nets, Gap Filling and Forests: A Global-Comparative Perspective. World Dev. 2014;64: S29–S42. doi: 10.1016/j.worlddev.2014.03.005

[33] Maes J, Liquete C, Teller A, Erhard M. An indicator framework for assessing ecosystem services in support of the EU Biodiversity Strategy to 2020 An indicator framework for assessing ecosystem services in support. Ecosyst Serv. 2016;17: 14–23. doi: 10.1016/j.ecoser.2015.10.023

[34] Pereira HM, Reyers B, Watanabe M. 8.Condition and Trends of Ecosystem Services and Biodiversity. Millenium Ecosyst Assess Multiscale Assess. 2005; 33.

[35] Balvanera P, Pfisterer AB, Buchmann N, He J-S, Nakashizuka T, Raffaelli D, et al Quantifying the evidence for biodiversity effects on ecosystem functioning and services. Ecol Lett. 2006;9: 1146–56. doi: 10.1111/j.1461-0248.2006.00963.x 16972878 10.1111/j.1461-0248.2006.00963.x

[36] Harrison PA, Berry PM, Simpson G, Haslett JR, Blicharska M, Bucur M, et al Linkages between biodiversity attributes and ecosystem services: A systematic review. Ecosyst Serv. 2014;9: 191–203. doi: 10.1016/j.ecoser.2014.05.006

[37] Raudsepp-Hearne C, Peterson GD, Tengö M, Bennett EM, Holland T, Benessaiah K, et al Untangling the Environmentalist’s Paradox: Why Is Human Well-being Increasing as Ecosystem Services Degrade? Bioscience. 2010;60: 576–589. doi: 10.1525/bio.2010.60.8.4

[38] Narayanasamy N. Participatory rural appraisal: Principles, methods and application SAGE Publications; 2009.

[39] Dazé A, Ambrose K, Ehrhart C. Climate Vulnerability and Capacity Analysis Handbook. CARE; 2009; 52.

[40] BPS. Badan Pusat Statistik Indonesia [Internet]. 2017 [cited 31 Jan 2017]. Available: www.bps.go.id

[41] Pearson T, Walker S, Brown S. Sourcebook for Land Use, Land-Use Change and Forestry Projects Winrock International and the BioCarbon Fund of the World Bank; 2005.

[42] Pearson TRH, Brown SL, Birdsey R a. Measurement Guidelines for the Sequestration of Forest Carbon [Internet]. General Technical Report NRS-18. Delaware: United States Department of Agriculture—Forest Service. 2007. doi: 10.1089/hum.2005.16.57

[43] Chave J, Réjou-Méchain M, Búrquez A, Chidumayo E, Colgan MS, Delitti WBC, et al Improved allometric models to estimate the aboveground biomass of tropical trees. Glob Chang Biol. 2014;20: 3177–3190. doi: 10.1111/gcb.12629 24817483 10.1111/gcb.12629

[44] ICRAF. Wood Density Database. In: World Agroforestry Center [Internet]. 2016 [cited 21 Jan 2015]. Available: http://www.worldagroforestry.org/sea/Prod-ucts/AFDbases/wd/Index.htm

[45] Hairiah K, Dewi S, Agus F, Velarde S, Ekadinata A, Rahayu S, et al Measuring Carbon Stocks Across Land Use Systems: a Manual [Internet]. World Agroforestry Centre 2010 Available: https://books.google.fr/books/about/Measuring_carbon_stocks.html?id=vWTxoAEACAAJ&redir_esc=y

[46] Michon G, Foresta H de, Levang P, Verdeaux F. Domestic forests: a new paradigm for integrating local communities´ forestry intro tropical forest science. Ecol Soc. 2007;12: 1 Available: http://www.ecologyandsociety.org/vol12/iss2/art1/

[47] van Noordwijk M, Bizard V, Wangpakapattanawong P, Tata HL, Villamor GB, Leimona B. Tree cover transitions and food security in Southeast Asia. Glob Food Sec. Elsevier; 2014;3: 200–208. doi: 10.1016/j.gfs.2014.10.005

[48] Sendzimir J, Reij CP, Magnuszewski P. Rebuilding Resilience in the Sahel: Regreening in the Maradi and Zinder Regions of Niger. Ecol Soc. 2011;16: 8 doi: 10.5751/ES-04198-160301

[49] Farley KA. Pathways to forest transition: Local case studies from the Ecuadorian Andes. 2010;9: 7–26.

[50] Labrière N, Laumonier Y, Locatelli B, Vieilledent G, Comptour M. Ecosystem Services and Biodiversity in a Rapidly Transforming Landscape in Northern Borneo. PLoS One. 2015;10: e0140423 doi: 10.1371/journal.pone.0140423 26466120 10.1371/journal.pone.0140423 PMC4605616

[51] Labrière N, Locatelli B, Vieilledent G, Kharisma S, Basuki I, Gond V, et al Spatial congruence between carbon and biodiversity across forest landscapes of northern Borneo. Glob Ecol Conserv. Elsevier; 2016;6: 105–120. doi: 10.1016/J.GECCO.2016.01.005

[52] Ravindranath NH. Mitigation and adaptation synergy in forest sector. Mitig Adapt Strateg Glob Chang. 2007;12: 843–853. doi: 10.1007/s11027-007-9102-9

[53] Locatelli B, Evans V, Wardell A, Andrade A, Vignola R. Forests and climate change in latin America: Linking adaptation and mitigation. Forests. Molecular Diversity Preservation International; 2011;2: 431–450. doi: 10.3390/f2010431

[54] Klein RJT, Midgley GF, Preston BL, Alam M, Berkhout FGH, Dow K, et al Adaptation Opportunities, Constraints, and Limits In: Field CB, editor. Climate Change 2014: Impacts, Adaptation, and Vulnerability Part A: Global and Sectoral Aspects. Cambridge: Cambridge University Press; 2014 pp. 899–943. doi: 10.1017/CBO9780511807756.003

[55] Moore ML, Tjornbo O, Enfors E, Knapp C, Hodbod J, Baggio JA, et al Studying the complexity of change: Toward an analytical framework for understanding deliberate social-ecological transformations. Ecol Soc. 2014;19 doi: 10.5751/ES-06966-190454

[56] Thoms CA. Community control of resources and the challenge of improving local livelihoods: A critical examination of community forestry in Nepal. Geoforum. 2008;39: 1452–1465. doi: 10.1016/j.geoforum.2008.01.006

[57] Armitage D. Adaptive capacity and community-based natural resource management [Internet]. Environmental Management. 2005 pp. 703–715. doi: 10.1007/s00267-004-0076-z 15940398 10.1007/s00267-004-0076-z

[58] Enfors E. Social-ecological traps and transformations in dryland agro-ecosystems: Using water system innovations to change the trajectory of development. Glob Environ Chang. 2013;23: 51–60. doi: 10.1016/j.gloenvcha.2012.10.007

[59] Carpenter SR, Folke C. Ecology for transformation [Internet]. Trends in Ecology and Evolution. 2006 pp. 309–315. doi: 10.1016/j.tree.2006.02.007 16769430 10.1016/j.tree.2006.02.007

[60] Folke C, Hahn T, Olsson P, Norberg J. Adaptive Governance of Social-Ecological Systems. Annu Rev Environ Resour. 2005;30: 441–473. doi: 10.1146/annurev.energy.30.050504.144511

[61] Pelling M. Learning from others: The scope and challenges for participatory disaster risk assessment. Disasters. 2007;31: 373–385. doi: 10.1111/j.1467-7717.2007.01014.x 18028159 10.1111/j.1467-7717.2007.01014.x

[62] Olsson P, Folke C, Berkes F. Adaptive Comanagement for Building Resilience in Social?Ecological Systems. Environ Manage. 2004;34: 75–90. doi: 10.1007/s00267-003-0101-7 15383875 10.1007/s00267-003-0101-7

[63] Biggs R, Carpenter SR, Brock WA. Turning back from the brink: detecting an impending regime shift in time to avert it. Proc Natl Acad Sci U S A. National Academy of Sciences; 2009;106: 826–31. doi: 10.1073/pnas.0811729106 19124774 10.1073/pnas.0811729106 PMC2630060

[64] Bakkegaard RK, Hogarth NJ, Bong IW, Bosselmann AS, Wunder S. Measuring forest and wild product contributions to household welfare: Testing a scalable household survey instrument in Indonesia. For Policy Econ. 2016; doi: 10.1016/j.forpol.2016.10.005

[65] Bong IW, Felker ME, Maryudi A. How are local people driving and affected by forest cover change? Opportunities for local participation in REDD+ Measurement, reporting and verification. Herold M, editor. PLoS One. Public Library of Science; 2016;11: e0145330 doi: 10.1371/journal.pone.0145330 27806044 10.1371/journal.pone.0145330 PMC5091836

[66] Peluso N. Rich forests, poor people: Resource control and resistance in Java—Peluso,NL. Ann Assoc Am Geogr. University of California Press; 1995;85: 748–750. doi: 10.2307/2059224

[67] Potter L. Agricultural Intensification in Indonesia: Outside Pressures and Indigenous Strategies. Asia Pac Viewp. Blackwell Publishers Ltd; 2001;42: 305–324. doi: 10.1111/1467-8373.00151

[68] Pelling M, Manuel-Navarrete D. From resilience to transformation: The adaptive cycle in two Mexican urban centers. Ecol Soc. 2011;16 doi: 10.5751/ES-04038-160211

[69] O’Brien. Global environmental change II: From adaptation to deliberate transformation Prog Hum Geogr. SAGE PublicationsSage UK: London, England; 2012;36: 667–676. doi: 10.1177/0309132511425767

[70] Gorddard R, Colloff MJ, Wise RM, Ware D, Dunlop M. Values, rules and knowledge: Adaptation as change in the decision context. Environ Sci Policy. Elsevier Ltd; 2016;57: 60–69. doi: 10.1016/j.envsci.2015.12.004

[71] Spangenberg JH, Görg C, Truong DT, Tekken V, Bustamante JV, Settele J. Provision of ecosystem services is determined by human agency, not ecosystem functions. Four case studies. Int J Biodivers Sci Ecosyst Serv Manag. 2014;10: 40–53. doi: 10.1080/21513732.2014.884166

[72] Dobusch L, Schüßler E. Theorizing path dependence: A review of positive feedback mechanisms in technology markets, regional clusters, and organizations. Ind Corp Chang. Oxford University Press; 2013;22: 617–647. doi: 10.1093/icc/dts029

[73] Wise RM, Fazey I, Stafford Smith M, Park SE, Eakin HC, Archer Van Garderen ERM, et al Reconceptualising adaptation to climate change as part of pathways of change and response. Glob Environ Chang. 2014;28: 325–336. doi: 10.1016/j.gloenvcha.2013.12.002

[74] Colloff MJ, Martín-López B, Lavorel S, Locatelli B, Gorddard R, Longaretti P-Y, et al An integrative research framework for enabling transformative adaptation. Environ Sci Policy. 2016;68: 10 doi: 10.1016/j.envsci.2016.11.007

[75] Matyas D, Pelling M. Positioning resilience for 2015: The role of resistance, incremental adjustment and transformation in disaster risk management policy. Disasters. 2015;39: s1–s18. doi: 10.1111/disa.12107 25494954 10.1111/disa.12107

[76] Feola G. Societal transformation in response to global environmental change: A review of emerging concepts. Ambio. 2015;44: 376–390. doi: 10.1007/s13280-014-0582-z 25431335 10.1007/s13280-014-0582-z PMC4510318

[77] Davies S. Are coping strategies a cop out? IDS Bull. 1993;24: 60–73.

[78] Kates RW, Travis WR, Wilbanks TJ. Transformational adaptation when incremental adaptations to climate change are insufficient. Proc Natl Acad Sci. National Academy of Sciences; 2012;109: 7156–7161. doi: 10.1073/pnas.1115521109 22509036 10.1073/pnas.1115521109 PMC3358899

[79] Kates RW, Travis WR, Wilbanks TJ. Transformational adaptation when incremental adaptations to climate change are insufficient. Proc Natl Acad Sci. 2012;109: 7156–7161. doi: 10.1073/pnas.1115521109 22509036 10.1073/pnas.1115521109 PMC3358899

[80] Olsson P, Galaz V, Boonstra WJ. Sustainability transformations: A resilience perspective. Ecol Soc. The Resilience Alliance; 2014;19: art1 doi: 10.5751/ES-06799-190401

[81] Thornton TF, Comberti C. Synergies and trade-offs between adaptation, mitigation and development. Clim Change. Springer Netherlands; 2017;140: 5–18. doi: 10.1007/s10584-013-0884-3

